# Design of dynamic adaptive control framework for exoskeleton robot driven by neuromuscular signal based on deep reinforcement learning

**DOI:** 10.3389/fncom.2026.1807958

**Published:** 2026-06-25

**Authors:** Kaidi Ma

**Affiliations:** AI Institute, Jiangsu XCMG National Key Laboratory Technology Co., Ltd., Xuzhou, Jiangsu, China

**Keywords:** deep reinforcement learning, exoskeleton robot, motion intention, motion intention modeling, neuromuscular signal

## Abstract

To enhance the dynamic adaptability and robustness of neuromuscular signal-driven exoskeleton robots in complex environments. This study develops a Deep Reinforcement Learning (DRL)-based exoskeleton control framework. A module for neuromuscular signal processing and motion intention modeling is designed, encompassing band-pass filtering, local normalization, time-frequency feature extraction, and Bidirectional Long Short-Term Memory (BiLSTM) time-series encoding. Additionally, a DRL-based dynamically adaptive control framework is established. Trajectory tracking error, torque smoothness, energy consumption agent, joint safety constraint and intention consistency are comprehensively introduced into the reward function to achieve the multi-objective balance of “accuracy-comfort-energy consumption-safety.” Comparative experiments demonstrate that the joint trajectory tracking errors of the proposed optimized system are 2.37°, 2.64°, and 2.92°, respectively. These values are significantly lower than those of comparative systems, including the Deep Reinforcement Learning-based Robust Controller for Lower-Limb Rehabilitation Exoskeletons (DRL-RC-LLRE) and the Learning-in-Simulation Exoskeleton Assistance Framework (LiS-EXO). The corresponding torque smoothness indicators are 0.62, 0.68, and 0.71. The mechanical energy consumption proxy indicators are 10.48, 11.39, and 11.67, indicating that while improving the tracking accuracy, the torque oscillation is effectively suppressed and the energy consumption is reduced. The ablation experiments further show that when any of the neuromuscular intention embedding, intention consistency reward or safety constraint modules are removed, the average trajectory error, torque smoothness and energy consumption indicators all deteriorate to varying degrees. The complete model achieves 2.63°, 0.66, and 11.17, respectively, in the three indicators, verifying the collaborative contribution of the three key modules to the overall performance. Overall, this study has constructed and verified a reproducible “neuromuscular signal-motion intention-reinforcement learning control” integrated framework, and thus having certain contributions to the field of intelligent exoskeleton control.

## 1 Introduction

Exoskeleton robot, as an important carrier of human–machine collaborative enhancement system, has been widely concerned in the fields of rehabilitation training, daily walking assistance and load reduction in industrial scenes in recent years (Luo et al., 2023; Samarakoon et al., 2025). Different from the traditional rigid mechanical structure, exoskeleton robot needs to be closely coupled with the human movement process in a complex and dynamic environment. Its control objectives include trajectory tracking and attitude stability, and the naturalness, comfort and long-term availability of movement (Huang et al., 2025; Toro-Ossaba et al., 2025). Therefore, how to realize the accurate perception of human motion intention and the dynamic adaptive adjustment of external skeleton control strategy has become one of the key scientific problems in this field (Akbaş et al., 2025). Among many signal sources, neuromuscular signals can reflect human muscle activation and exercise intention in a forward-looking way in time, which is an important information channel for constructing “man–machine-environment” closed-loop control (Sul et al., 2025; Zhang et al., 2024). At present, a variety of published data sets of neuromuscular signals and gait provide rich experimental basis for related research, enabling researchers to systematically model and analyze different motion patterns, joint behaviors and muscle activation patterns without involving real subjects’ experiments and ethical issues (Wang and Luo, 2025; Acuña Luna et al., 2025). However, this kind of signal has the characteristics of high noise, strong nonlinearity and significant individual/task difference. It is difficult to map to high-level motion intention or joint reference trajectory stably and reliably in complex tasks and multiple scenes only by relying on traditional linear models or simple threshold methods (Khan, 2025). On the other hand, the existing exoskeleton control methods are mostly based on preset trajectory control, impedance/force control or finite state machine, and often rely on artificially designed control laws and prior experience (Zhang et al., 2025). In the face of terrain change, load change or Human-Computer Interaction (HCI) mode change, this method has limited adaptability and generalization ability. It is difficult to update the strategy in time according to the combined change of “human intention-system state-environmental disturbance” (Nasr et al., 2024; Van der Kooij et al., 2025).

Therefore, this study focuses on the design of a dynamically adaptive control framework for exoskeleton robots, aiming to establish a relatively comprehensive technical pathway and verification system encompassing three key aspects: theoretical modeling, algorithm design, and simulation experiments.

## 2 Literature review

Neuromuscular signals serve as an important information source reflecting human motion intention in exoskeleton control (Sedighi et al., 2023). In recent years, numerous studies have investigated different signal preprocessing and feature extraction methods based on public datasets or motion capture datasets to improve the accuracy of motion recognition and motion intention prediction (Xiong et al., 2024; Rad and Brisan, 2025). Among them, model-free myoelectric control methods based on statistical features, pattern recognition, and deep convolutional neural networks have been widely applied in exoskeleton control research (Shakeriaski and Mohammadian, 2025; Kerdjidj et al., 2023). However, most of these studies only focus on signal processing or classification tasks themselves, and have not yet formed deep coupling with control systems (Müller, 2024). Related studies have found that dynamic motion recognition accuracy can be effectively improved by performing time-domain feature extraction and pattern classification on public datasets. They adopted traditional features including Root Mean Square (RMS), Zero Crossing (ZC), and Mean Absolute Value (MAV) combined with the Support Vector Machine (SVM) classifier to achieve high-precision recognition of multiple motion categories (Song et al., 2023). Nevertheless, this method relies heavily on manual feature engineering. Existing studies have shown that the robustness and generalization ability of traditional handcrafted features are easily limited when dealing with complex nonlinear and time-varying motion patterns as well as cross-condition signal distribution changes (Li et al., 2024; Zheng et al., 2025), thus making it difficult to be directly applied to real-time exoskeleton control pipelines.

Given its strengths in high-dimensional continuous control scenarios, Deep Reinforcement Learning (DRL) has increasingly become a focal point of research in the field of exoskeleton control. Related work mainly focuses on strategy learning, trajectory tracking and environmental adaptability. However, most studies are still limited by the lack of model coupling, limited generalization ability or incomplete control framework (Wang et al., 2025). Drewing et al. (2024) found that the exoskeleton control strategy of lower limbs with good tracking performance can be trained by using Proximal Policy Optimization (PPO) DRL algorithm in the simulation environment. Experimental results show that DRL control can adapt to different gait speeds. However, its control strategy is not integrated with neuromuscular signal data, and only depends on the joint state, so it is difficult to realize the cooperative control structure of “man–machine joint decision-making.”

Motion intention recognition is the core link of neuromuscular signal-driven exoskeleton control, and its goal is to extract high-level semantic information from EMG data or gait data, such as motion category, joint target value or gait stage (Hashemi et al., 2023). Related research has made rapid progress in deep learning algorithm, but it still faces problems such as weak generalization ability and separation from control module (Jung et al., 2025). Sharifi-Renani et al. (2023) found that by constructing the gait phase prediction model based on Transformer, the motion sequence can be accurately reconstructed on the public gait dataset, and the joint angles in several future time steps can be predicted. Although this method improves the prediction accuracy, it does not further discuss how to use the prediction results for closed-loop control of exoskeleton, nor does it verify the generalization ability in multi-task scenes.

Therefore, this study proposes an integrated framework of “signal-intention-control” based on public neuromuscular datasets, forming a complete pipeline from signal representation and motion intention modeling to DRL control output. It should be noted that the feature vector constructed in this study is not used as the final discriminative basis to replace deep strategy learning, but as part of the state description, which together with motion intention variables and exoskeleton dynamic states constitutes the input of the control policy network. Different from traditional handcrafted features mainly oriented to offline classification tasks, the feature vector in this study aims to improve the effectiveness of multi-source information organization and state representation, and realize dynamic decision optimization through the subsequent reinforcement learning process. On this basis, this study further constructs a DRL dynamic adaptive control framework for complex environments, and enhances the robustness and generalization ability of the policy in complex scenarios through multi-disturbance training and domain randomization design.

## 3 Research method

### 3.1 Design of exoskeleton control system driven by neuromuscular signal

**Figure 1:**
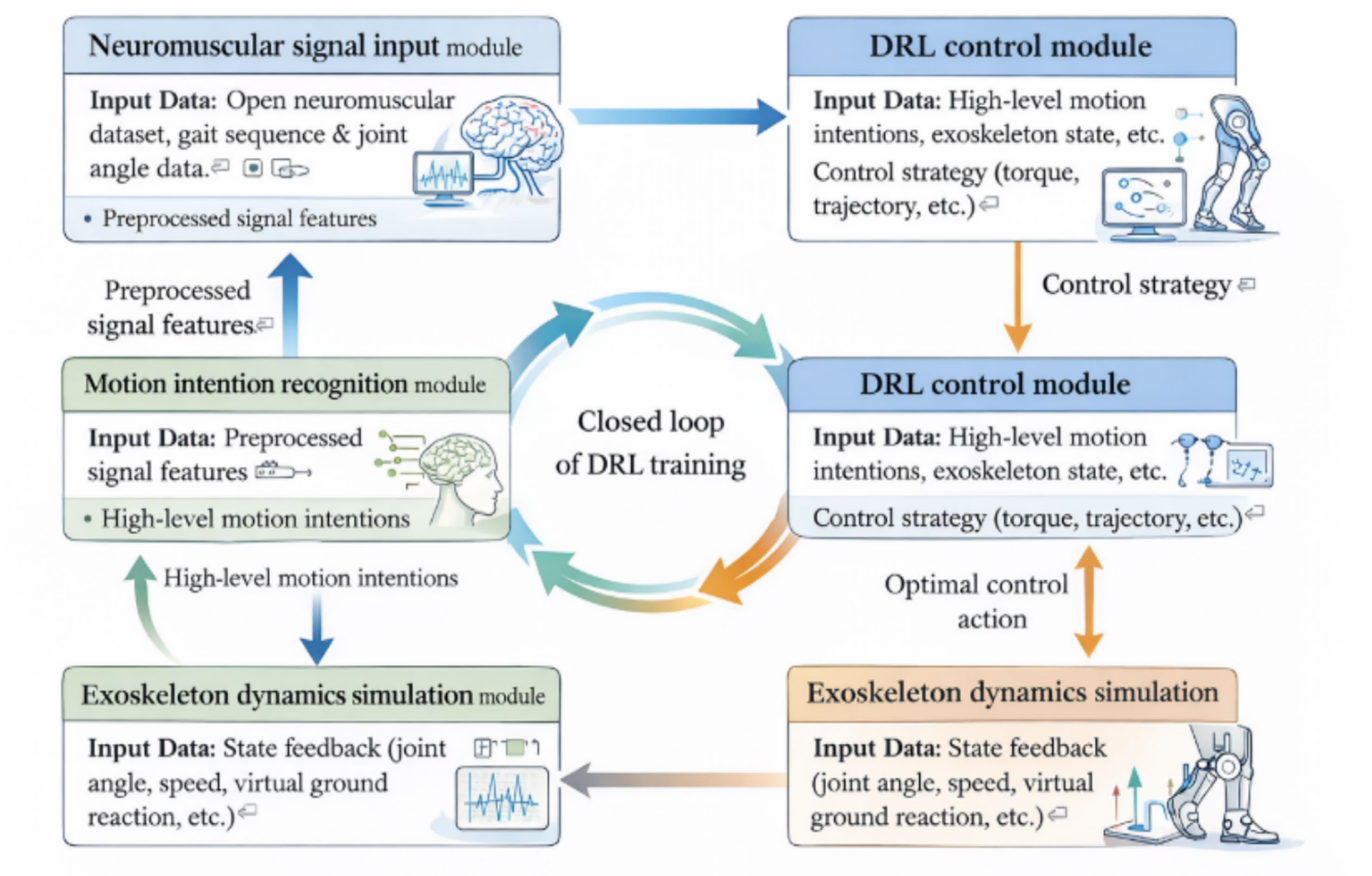
Functions, inputs, and outputs of each module of the exoskeleton control system driven by neuromuscular signals.

The core goal of neuromuscular signal-driven exoskeleton control system is to realize the complete mapping process from neuromuscular signal characteristics obtained from public data sets to exoskeleton control instructions (Yousuf, 2025; Abdallah et al., 2025). To ensure the reproducibility and expansibility of the system, a four-level structure including “signal input layer-intention modeling layer-reinforcement learning control layer-exoskeleton simulation layer” is designed based on the data set and simulation environment. The system structure is dominated by neuromuscular signals, and the DRL strategy network is driven by high-level motion intention variables, thus forming a dynamic adaptive exoskeleton control framework with closed loop of perception-decision-execution, as shown in Figure 1.

From the perspective of the overall system architecture, this study constructs a four-level control framework consisting of signal input, motion intention recognition, DRL control, and exoskeleton dynamics feedback, realizing a complete closed loop from neuromuscular signal processing to control command output. Different from traditional methods that only use neuromuscular signals for motion classification, this study further converts them into intention variables that can characterize motion trends and control requirements, and inputs them together with the exoskeleton dynamics state into the policy network. Based on this joint modeling method, the system can learn control strategies that take into account foresight, stability and robustness during simulation training, thereby achieving more effective dynamic adaptive adjustment in complex scenarios such as terrain changes, noise disturbances and task switching.

### 3.2 Neuromuscular signal processing and motion intention modeling

A complete modeling process of “original signal → time-frequency characteristics → time-series coding → motion intention” is studied and constructed. Consider the neuromuscular signals of C channels contained in the public dataset, and remember that the original discrete signal of the c channel is $x_{c}$ . First, it is subjected to band-pass filtering and intra-window normalization to obtain the preprocessed signal ${\tilde{y}}_{c}(k,n)$ , which is defined as Equation 1:


$${\tilde{y}}_{c}(k,n)=\frac{\sum_{m=0}^{M-1}\;h_{\mathrm{bp}}(m)x_{c}(\mathrm{kH}+n-m)-\mu_{c}(k)}{\sigma_{c}(k)+\varepsilon },n=0,\ldots ,L-1$$ (1)

k is the index of time window. n is the index of sampling points in current time window. $h_{\mathrm{bp}}$ is the impulse response sequence of bandpass filter. M is the length of bandpass filter. H is the step size of sliding window. L is the number of sampling points in each time window. $\mu_{c}(k)$ is the average value in time window. $\sigma_{c}(k)$ is the standard deviation in time window, ε is a numerical stability constant introduced to prevent the denominator from approaching zero. It shows that neuromuscular signals are often simultaneously affected by baseline drift, high-frequency noise, cross-channel amplitude differences, and statistical distribution fluctuations across time windows. Bandpass filtering is employed to retain the main frequency band components related to muscle activation and gait variation, while intra-window normalization is used to reduce amplitude scale differences among different individuals, acquisition sessions, and muscle groups, thereby improving the stability of subsequent feature extraction. Based on preprocessing signals, this study describes the time-frequency characteristics of neuromuscular signals from two aspects: short-term energy and spectral centroid. For each channel in the k th time window, the short-term energy sum and spectral centroid are defined as Equations 2–4:


$$E_{c}(k)=\sum_{n=0}^{L-1}\;{|{\tilde{y}}_{c}(k,n)w(n)|}^{2}$$ (2)

$$\Phi_{c}(k)=\frac{\sum_{l=0}^{F-1}\omega_{l}{|S_{c}(k,\omega_{l})|}^{2}}{\sum_{l=0}^{F-1}{|S_{c}(k,\omega_{l})|}^{2}}$$ (3)

$$S_{c}(k,\omega_{l})=\sum_{n=0}^{L-1}\;{\tilde{y}}_{c}(k,n)w(n)e^{-j\omega_{l}n}$$ (4)

$E_{c}(k)$ is a short-time energy feature. $\Phi_{c}(k)$ is a spectral centroid feature. w(n) is a windowing function. F is the number of discrete frequency sampling points. $\omega_{l}$ is a discrete angular frequency sampling point. $S_{c}(k,\omega_{l})$ is a short-time Fourier transform coefficient, and j is an imaginary unit. Short-time energy can directly characterize the dynamic changes in muscle activation intensity and motion exertion level within a local time window, and exhibits high sensitivity to distinguishing different movement phases and diverse assistance requirements. Spectral centroid reflects the “center of gravity” position of signal energy in the frequency domain, which can be used to characterize muscle contraction patterns, gait transitions, and variations in frequency structure. Given that neuromuscular signals possess obvious non-stationarity and time-variability, relying solely on a single type of temporal or frequency-domain description is often insufficient to fully represent the motion process. Therefore, this study combines short-time energy and spectral centroid to retain both amplitude dynamics and frequency distribution information simultaneously with a low feature dimension. The introduction of the short-time Fourier transform coefficient $S_{c}(k,\omega_{l})$ provides a unified local frequency spectrum representation for the calculation of the spectral centroid. After the energy and spectral centroid of all channels are obtained, they are spliced to form the feature vector corresponding to the k th time window, as expressed in Equation 5:


$$z_{k}={\left[E_{1}(k),\ldots ,E_{C}(k),\Phi_{1}(k),\ldots ,\Phi_{C}(k)\right]}^{⊤}$$ (5)

$z_{k}$ is a comprehensive feature vector. This feature vector is not intended to directly replace subsequent deep decision-making learning, but serves as part of the basic state representation. It is used to compress multi-channel neuromuscular signals into an input representation with clear physiological significance and time-frequency interpretability, thereby providing a stable input for subsequent temporal coding and motion intention modeling. The feature sequence is encoded using Equations 6 and 7:


$${\vec{h}}_{k}=f_{\text{LSTM}}(z_{k},{\vec{h}}_{k-1};\theta_{f})$$ (6)

$${\overset{\leftarrow }{h}}_{k}=f_{\text{LSTM}}(z_{k},{\overset{\leftarrow }{h}}_{k+1};\theta_{b})$$ (7)

${\vec{h}}_{k}$ is the hidden state vector of forward Long Short-Term Memory (LSTM) at time step k . ${\overset{\leftarrow }{h}}_{k}$ is the hidden state vector of backward LSTM at time step k . $f_{\text{LSTM}}$ is the nonlinear state updating function of LSTM cell. ${\vec{h}}_{k-1}$ and ${\overset{\leftarrow }{h}}_{k+1}$ are the hidden states of the previous/subsequent time step. $\theta_{f}$ and $\theta_{b}$ are parameter sets. Furthermore, this study concatenates the bidirectional hidden states to obtain a temporal representation vector, which is used as a shared representation for the subsequent joint prediction of discrete motion intentions and continuous motion intentions. The motivation for adopting BiLSTM is that unidirectional temporal models mainly rely on historical information, whereas the bidirectional structure can utilize both forward and backward contextual dynamic clues simultaneously, which is more conducive to capturing the contextual dependencies in gait phase transitions, motion switching, and joint trend variations.

In the study of obtaining time series coding, the multi-task learning method is used to predict both discrete motion intentions (such as motion category and gait stage) and continuous motion intentions (such as expected joint angle vector). The multi-task loss function is defined as Equation 8:


$$\begin{array}{l} L=\frac{1}{N}\sum_{k=1}^{N}[{\alpha \mathrm{CE}(p_{k},{\hat{p}}_{k})+\beta q_{k}-{\hat{q}}_{k}\|}_{2}^{2}]\\ {+\frac{\gamma }{N-1}\sum_{k=2}^{N}∥{\hat{q}}_{k}-{\hat{q}}_{k-1}\|}_{2}^{2}+\lambda \Theta_{2}^{2} \end{array}$$ (8)

ℒ is the training loss function. N is the total number of time windows. $p_{k}$ is the real category one-hot vector. ${\hat{p}}_{k}$ is the prediction category probability vector. $q_{k}$ is the real continuous motion intention vector. ${\hat{q}}_{k}$ is the prediction continuous motion intention vector. CE is the cross-entropy loss function. β and α are the importance coefficients. γ is the coefficient for controlling the time smoothness of continuous intention output. λ is the regularization coefficient, and Θ is the trainable parameter set. Through the above modeling pipeline, this study realizes hierarchical representation from neuromuscular signal preprocessing, time-frequency feature extraction, and temporal context modeling to joint prediction of discrete and continuous motion intentions, providing interpretable and temporally consistent intention inputs for the subsequent DRL controller.

### 3.3 Dynamic adaptive control framework based on DRL

In this study, the exoskeleton control problem is formalized as a discrete-time Markov Decision Process (MDP). In time t , the agent generates actions according to the current state $s_{t}$ , and obtains the immediate return $r_{t}$ and the next state in the simulation environment. To model the movement intention and exoskeleton dynamics uniformly, this study constructs the state vector as follows Equation 9:


$$s_{t}={\left[\psi_{\mathrm{int}}(h_{t}),x_{t},\xi_{t}\right]}^{⊤}$$ (9)

$\psi_{\mathrm{int}}(h_{t})$ is a nonlinear mapping function. $\xi_{t}$ is an environment and task parameter vector. $x_{t}$ is a dynamic state vector. The reason for adopting such a separate modeling approach is that directly feeding high-dimensional temporal coding results into the policy network tends to cause state redundancy and training instability. By forming an intention embedding of unified dimension via $\psi_{\mathrm{int}}(h_{t})$ , the convergence of policy learning and the consistency of state representation can be improved while retaining key temporal information.

To guide the DRL strategy to learn a control behavior that considers trajectory tracking accuracy, control smoothness, energy consumption proxy and safety constraints, the following multi-objective weighted reward functions are designed in this study. Let the target joint angle be $q_{t}^{⋆}$ , the current joint angle be $q_{t}$ , and the control output joint torque be $\tau_{t}$ , then the instant reward for each time step is defined as Equation 10:


$$\begin{array}{l} r_{t}=-w_{1}q_{t}-{q_{t}^{⋆}}_{2}^{2}-w_{2}{\tau_{t}}_{2}^{2}-w_{3}\tau_{t}-{\tau_{t-1}}_{2}^{2}\\ -w_{4}1_{\left\{q_{t}\notin Q_{\text{safe}}\right\}}-w_{5}1_{\left\{∥{\dot{q}}_{t}{∥}_{2}>v_{\max}\right\}}+w_{6}\rho_{t} \end{array}$$ (10)

${\dot{q}}_{t}$ is the joint angular velocity vector. $Q_{\text{safe}}$ is the set of safe working intervals of joint angles. $v_{\max}$ is the allowable maximum joint angular velocity threshold. $\rho_{t}$ is the measure of intention consistency. $w_{1}-w_{6}$ is the non-negative weight coefficient. Based on the given reward function, this study adopts the strategy with entropy regularization to optimize the target to enhance the exploration ability and robustness of the strategy. Among them, the intention consistency metric is used to characterize the degree of consistency between the control behavior and the current motion intention embedding, thereby encouraging the control strategy to be as consistent as possible with the user’s motion trend inferred from neuromuscular signals while satisfying dynamic constraints. To avoid training instability caused by excessive differences in magnitudes among different reward terms, this study adopts weight balancing for each reward sub-term and batch normalization for the total return.

The strategy network is $\pi_{\theta }(\cdot |s_{t})$ , the discount factor is γ , and the entropy regularization coefficient is η , then the objective function is defined as Equation 11:


$$J(\theta )=E_{\pi_{\theta }}[\sum_{t=0}^{T-1}\;\gamma^{t}(r_{t}+\mathrm{\eta ℋ}(\pi_{\theta }(\cdot |s_{t})))]$$ (11)

J(θ) is the objective function of expected return. $E_{\pi_{\theta }}$ is the mathematical expectation of trajectory under the state-action distribution. ℋ is Shannon entropy. To improve the training efficiency and stability, this study adopts the actor-critic structure and combines the strategy network with the value network for joint optimization. Let the value network $V_{\phi }(s_{t})$ approximate the current discount return expectation, and the corresponding advantage function is defined as Equation 12:


$$A_{t}=(\sum_{k=0}^{K-1}\;\gamma^{k}r_{t+k}+\gamma^{K}V_{\phi }(s_{t+K}))-V_{\phi }(s_{t})$$ (12)

$A_{t}$ is the dominance function. K is the number of steps used to construct the multi-step return, and ϕ is the parameter set of the value network. In practical training, the policy network updates parameters by maximizing the log policy probability weighted by the advantage function, while the value network learns by minimizing the mean squared error between multi-step returns and state value estimates. For each training batch, this study standardizes the cumulative returns or advantage values to reduce fluctuations caused by differences in trajectory lengths and reward scales during gradient updates. This processing is only used to improve numerical stability and training reproducibility, without altering the physical meaning of the reward function itself.

## 4 Experimental design

In this study, the experiments adopt the public dataset A Human Lower-Limb Multimodal Dataset under Diverse Conditions for Facilitating Rehabilitation Robotics (K2MUSE). The dataset was collected in a biomechanical laboratory environment and contains lower-limb multimodal motion data from 30 healthy subjects aged 22–34 years, including 20 males and 10 females. Data collection covers various terrain conditions, including level ground (0°), ramps of ±5° and ±10°, as well as three walking speeds: 0.5 m/s, 1.0 m/s, and 1.5 m/s. Non-ideal acquisition scenarios, such as muscle fatigue, electrode shift, and inter-day collection differences, are also taken into account. Kinematic data were captured by 8 Vicon V5 optical motion capture cameras at 100 Hz, and marker placement follows the Vicon Plug-in Gait lower-limb model, from which lower-limb joint kinematics and dynamics parameters are derived. The treadmill system is a Bertec instrumented treadmill with dual force plates, which records ground reaction forces and related dynamic information at 1000 Hz. Surface electromyography (sEMG) signals are collected by the Noraxon Ultium EMG system at 2000 Hz. The recorded muscles include the right tibialis anterior, medial gastrocnemius, lateral gastrocnemius, soleus, rectus femoris, vastus lateralis, vastus medialis, biceps femoris, semitendinosus, as well as the left tibialis anterior, lateral gastrocnemius, rectus femoris, and biceps femoris, covering a total of 13 muscles in both lower limbs. The dataset also synchronously recorded A-mode ultrasound signals of the left tibialis anterior, lateral gastrocnemius, rectus femoris, and biceps femoris to characterize muscle thickness and deformation changes. For joint variables, the dataset mainly provides ankle, knee, and hip joint angles, torques, and related dynamic parameters calculated based on the Plug-in Gait model. The dataset can be downloaded from https://arxiv.org/abs/2504.14602.

The experiments of this study are conducted on a unified software and hardware platform. The computing device is an NVIDIA RTX 4090 graphics card with a 24 GB memory capacity. The central processing unit is an Intel Core i9-13900K with a base frequency of 3.0 GHz. The system memory is 64 GB DDR5. The experimental operating system is Windows 11 Pro, version 22H2. The training of the deep learning model relied on the PyTorch framework, version 2.2.1, and CUDA 12.1 is used as the GPU acceleration backend. The reinforcement learning part is implemented based on the Stable-Baselines3 framework, with a Python environment version of Python 3.10. The exoskeleton simulation experiment is built on the Mujoco 2.3.5 dynamics engine and data interaction with the DRL training module is achieved through a custom interface. In addition, to ensure the stability of training and the reproducibility of the experiment, parameters are set. The time window length is set to 200 ms, and the sliding step is 20 ms. The electromyographic signals are input into the feature extraction network after being filtered by a 20–500 Hz band-pass filter. The feature encoding used two layers of BiLSTM, with 128 hidden units in each layer, and the output intent embedding dimension is 64. This study adopts the Soft Actor-Critic algorithm as the main training framework. Both the policy network and the value network consist of three fully connected layers, with 256, 256, and 128 neurons in each layer, respectively. The activation function adopted is ReLU. For the experiments, two comparative systems are selected: the DRL-based Robust Controller for Lower-Limb Rehabilitation Exoskeletons (DRL-RC-LLRE) and the Learning-in-Simulation Exoskeleton Assistance Framework (LiS-EXO). In this study, DRL-RC-LLRE and LiS-EXO are selected as comparative systems, mainly because they represent two typical research paradigms: robust reinforcement learning-based exoskeleton control and simulation learning-driven exoskeleton assistance generation, respectively. DRL-RC-LLRE focuses on learning robust walking control policies in musculoskeletal coupled simulations, with emphasis on trajectory tracking and stable control of the exoskeleton under uncertain interaction conditions. LiS-EXO concentrates on learning cross-activity assistance strategies in high-fidelity human-exoskeleton simulations, highlighting assistance generation and sim-to-real transfer without real human experiments. In contrast, the proposed method places stronger emphasis on motion intention modeling driven by neuromuscular signals. It integrates physiological signal features, intention representation, and dynamic states into a unified reinforcement learning control framework, forming a closed-loop pipeline of “signal-intention-control.”

The experimental validation in this study is mainly carried out in an exoskeleton simulation environment built based on the MuJoCo dynamics engine. Its purpose is to evaluate the relative differences of different control strategies in terms of trajectory tracking, smoothness, energy consumption proxy, robustness, and generalization under controllable and repeatable conditions. This setup effectively supports performance comparisons at the algorithmic level. However, the results in this study mainly reflect the control performance under simulation conditions and do not cover factors that may arise during real-world hardware deployment, such as sensing delay, actuator nonlinearity, human-machine contact error, and individual differences in wearing. Therefore, the conclusion regarding performance improvement in this study should be interpreted as verification of the simulation effectiveness of the proposed method, rather than the final proof of its application effect on real exoskeleton systems.

## 5 Experimental comparison and result analysis

### 5.1 Performance comparison experiment

**Figure 2:**
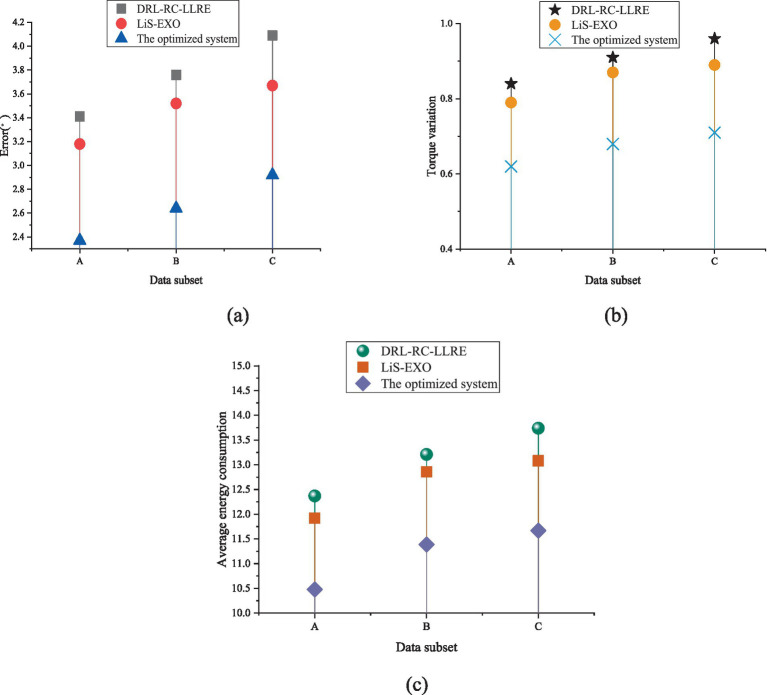
Performance comparison results. **(a)** Joint tracking error, **(b)** torque smoothness, and **(c)** mechanical energy consumption proxy.

To evaluate the effectiveness of the proposed DRL-based exoskeleton control framework, this section conducts comparative analyses of three key metrics using data subsets A, B, and C: joint trajectory tracking error, torque smoothness, and mechanical energy consumption proxy indicator. The results are presented in Figure 2.

Based on the joint trajectory tracking error metric in Figure 2, the proposed optimized system achieves errors of 2.37° (Subset A), 2.64° (Subset B), and 2.92° (Subset C) across the three data subsets, with the overall error level maintained at approximately 3°. Compared with DRL-RC-LLRE, the error in subset A is 3.41, while the error in the same subset of LiS-EXO is 3.18. It shows that the proposed optimized system achieves smaller trajectory deviation under the same data conditions. With the task difficulty gradually increasing from subset A to subset C, the errors of the three systems have increased, but the error growth rate of the proposed optimized system is relatively small, indicating that the tracking ability degradation is more moderate under complex conditions. In terms of torque smoothness, the RMS of the torque variation of the optimized system on three data subsets A, B and C are 0.62, 0.68, and 0.71, respectively. Compared with results of DRL-RC-LLRE on subset A of 0.84 and LiS-EXO on subsets B and C of 0.89, the control torque output by the proposed optimized system is smoother as a whole. Especially in the scene of data subset C, which contains more disturbances and various gait patterns, the torque fluctuation level of the optimized system is still maintained at about 0.71, showing good control stability and compliance. On the proxy index of mechanical energy consumption, the average energy consumption proxy of the optimized system on data subsets A, B, and C is 10.48, 11.39, and 11.67, respectively. Compared with DRL-RC-LLRE on subset C of 13.74 and LiS-EXO on subset B of 12.86, it shows that the optimized system effectively reduces the energy consumption corresponding to the combination of joint torque and angular velocity. As tasks transition from Subset A to Subset C, the energy consumption of all three systems shows an upward trend. However, the energy consumption curve of the optimized system consistently remains the lowest among the three. This indicates that its control strategy is more energy-efficient during behavioral execution. To avoid statistical inference based solely on single-run results on data subsets A, B, and C, this study performs multiple independent repeated experiments for each control system on every data subset. Repeated samples are obtained by varying random initialization, perturbation injection order, and training random seeds. On this basis, paired t-tests are conducted to evaluate the performance differences between different systems, using the metric results from repeated experiments under each subset as statistical samples. The results are presented in Table 1.

**Table 1:** Significance test results.

Metric	Comparison	*p*-value	Significant (*α* = 0.05)
Joint Tracking Error	The proposed optimized system vs. DRL-RC-LLRE	0.01	Yes
	The proposed optimized system vs. LiS-EXO	0.02	Yes
Torque Smoothness	The proposed optimized system vs. DRL-RC-LLRE	0.03	Yes
	The proposed optimized system vs. LiS-EXO	0.04	Yes
Mechanical Energy Consumption Proxy	The proposed optimized system vs. DRL-RC-LLRE	0.02	Yes
	The proposed optimized system vs. LiS-EXO	0.03	Yes

From Table 1, in terms of joint trajectory tracking error, torque smoothness and mechanical energy consumption proxy, the performance improvement of the proposed optimized system has passed the significant test compared with DRL-RC-LLRE and LiS-EXO. All *p* values are less than 0.05, indicating that the advantages of the proposed optimized system in control accuracy, control stability and energy consumption efficiency are not accidental fluctuations, but substantial improvements with statistical significance.

### 5.2 Experiment of robustness and generalization performance evaluation

**Figure 3:**
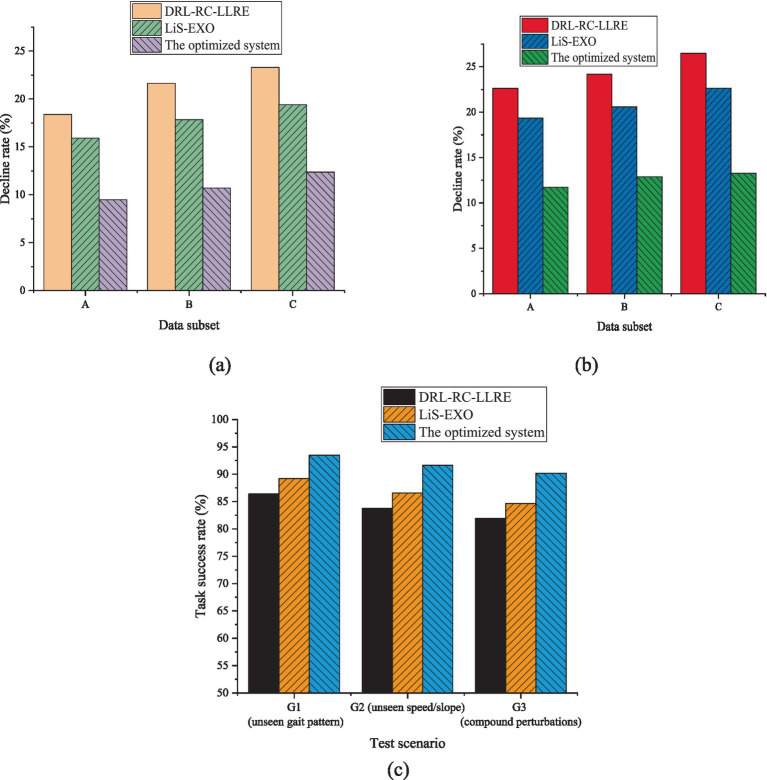
Comparison results of robustness and generalization performance. **(a)** Robustness under signal noise disturbance **(b)** robustness under electrode shift disturbance **(c)** generalization performance in unseen task conditions.

After the comparison of basic performance, to further verify the stability and generalization ability of different control systems under the conditions of sensor disturbance and task change, the study evaluates DRL-RC-LLRE, LiS-EXO and the proposed optimized system from two aspects of “robustness” and “generalization,” and the results are shown in Figure 3.

The results in Figure 3 show that, in terms of robustness under signal noise disturbance, the performance degradation rate of the proposed optimized system is 9.48, 10.71 and 12.36% on data subsets A, B and C, respectively. The overall performance remains in a relatively low range and rises steadily with task complexity. In contrast, in subset A, the decline rate of DRL-RC-LLRE is 18.37%, and that of LiS-EXO is 15.92%, which is obviously higher than that of the proposed optimized system in the same subset. On subset C, the performance of DRL-RC-LLRE is further reduced to 23.29%, and that of LiS-EXO is also increased to 19.41%. While the proposed optimized system is still controlled at 12.36% under this subset, showing obvious advantages in noise disturbance scenarios. In the electrode offset disturbance scenario, the performance degradation rates of the proposed optimized system on data subsets A, B, and C are 11.73, 12.89, and 13.26%, respectively. With the electrode position gradually shifting from the ideal attachment, the performance of the three systems has deteriorated, but the decline rate of DRL-RC-LLRE on subset C has reached 26.47%, and that of LiS-EXO is also 22.63%, which is significantly higher than that of the proposed optimized system (13.26%) under the same conditions. Even in subset B with moderate difficulty, the decline rate of the proposed optimized system is 12.89%, which is still smaller than that of DRL-RC-LLRE (24.18%) and LiS-EXO (20.57%), which shows that the proposed optimized system is less sensitive to the change of input distribution when structural disturbance such as electrode offset occurs in the sensing layer. In terms of cross-domain generalization ability, the proposed optimized system’s task success rates are 93.47, 91.62, and 90.18% in no gait combination scene G1, no speed/slope scene G2 and compound disturbance scene G3, respectively. In contrast, the success rate of DRL-RC-LLRE in G1 scene is 86.39%, and that of LiS-EXO is 89.21. In G2 scene, DRL-RC-LLRE and LiS-EXO reaches 83.74 and 86.57% respectively, which are lower than 91.62% of the proposed optimized system in the same scene. In G3 scene with the most complicated superposition of tasks and disturbances, the success rate of DRL-RC-LLRE is 81.92%, and that of LiS-EXO is 84.63%, while the proposed optimized system can still be maintained at the level of 90.18%. It shows that it still has strong task completion ability without the combination of tasks and complex disturbances. To verify the statistical significance of the proposed optimized system’s performance advantages in robustness and generalization scenarios, this study conducts analyses based on multiple independent experimental results under three disturbance conditions: noise interference, electrode displacement, and cross-domain generalization. The results are presented in Table 2.

**Table 2:** Significance test.

Metric/condition	Comparison	*p*-value	Significant (*α* = 0.05)
Noise robustness	The proposed optimized system vs. DRL-RC-LLRE	0.014	Yes
	The proposed optimized system vs. LiS-EXO	0.021	Yes
Electrode-shift robustness	The proposed optimized system vs. DRL-RC-LLRE	0.017	Yes
	The proposed optimized system vs. LiS-EXO	0.028	Yes
Generalization to unseen task scenario	The proposed optimized system vs. DRL-RC-LLRE	0.008	Yes
	The proposed optimized system vs. LiS-EXO	0.015	Yes

The statistical test results show that the proposed optimized system is significantly different from DRL-RC-LLRE and LiS-EXO under three conditions: noise disturbance, electrode offset disturbance and cross-domain generalization, and all *p* values are less than 0.05. This shows that its performance advantage under complex disturbance and no task conditions is not accidental fluctuation, but a statistically significant steady improvement.

### 5.3 Ablation experiment

To further analyze the contribution of key modules in the proposed optimized system to overall performance, an ablation experiment is designed in this section. First, different functional components are stripped from the framework. Subsequently, various model variants are constructed. Finally, these variants are compared under the same data subset and evaluation indicators. The evaluation indicators include joint trajectory tracking error, torque smoothness, and mechanical energy consumption proxy. Specifically, it includes the following four configurations:

1) Full model: the proposed optimized system (complete frame);
2) w/o Intent Embedding: remove the intention embedding of neuromuscular signals, and only use low-level dynamic information such as joint state to construct state vectors;
3) w/o Intention Consistency Reward: keep intention embedding and security constraints, but remove the items related to intention consistency from the reward function;
4) w/o Safety Constraints: remove safety penalties such as joint crossing and excessive speed, and only keep trajectory error, energy consumption proxy and basic smoothness constraints.

The experimental results are displayed in Table 3.

**Table 3:** Results of ablation experiment.

Model variant	Joint tracking error (°)	Torque smoothness (RMS)	Energy proxy
Full model	2.63	0.66	11.17
w/o Intent Embedding	3.21	0.71	11.83
w/o Intention Consistency Reward	2.92	0.81	12.09
w/o Safety Constraints	2.73	0.93	12.47

Experimental results indicate that the proposed optimized system delivers the best performance in the three indicators: the joint trajectory tracking error is 2.63, the torque smoothness is 0.66, and the energy consumption proxy index is 11.17. When the neuromuscular intent embedding module (w/o Intent Embedding) is removed, the error rises to 3.21, and the energy consumption also increases, indicating that it is difficult to make full use of the prospective intent information contained in neuromuscular signals only depending on the joint state. When the w/o Intention Consistency Reward is removed, the trajectory error is still better than the variant without intention embedding, but the torque smoothness is obviously worse, the RMS rises to 0.81, and the energy consumption is also increased. This indicates that the intention consistency term serves a pivotal role in constraining excessive strategy adjustments and enhancing the naturalness of control trajectories. After removing the safety constraints (w/o Safety Constraints), although the average trajectory error remains at a low level, the torque smoothness is the worst and the energy consumption is the highest. It shows that the strategy will exchange “radical control” for the reduction of local errors when there are no safety boundary and constraints, which is unreasonable from the perspective of overall system performance and deployment.

### 5.4 Ablation study on temporal encoders

To further analyze the influence of different temporal encoding structures on motion intention modeling and control performance, this study conducts comparative experiments between BiLSTM and Transformer Encoder under consistent settings of input features, training data split, DRL control framework, and other hyperparameters. Both encoders take the previously constructed time-frequency feature sequences as input and output motion intention representations with unified dimensions, which are then fed into the same DRL control module to ensure fair comparison. Specifically, BiLSTM performs joint modeling of information from forward and backward time steps through a bidirectional recurrent structure, which can effectively capture local temporal dependencies and contextual variations. In contrast, Transformer Encoder models global sequence relationships relying on the multi-head self-attention mechanism, exhibiting strong representation ability for long-range dependencies. Considering that neuromuscular signals and their derived time-frequency features present obvious temporal continuity in motion transition, gait variation and muscle activation patterns, comparing the practical performance of the two encoders on the current task helps further evaluate the adaptability of the temporal modeling module. The experiments are still conducted on data subsets A, B and C, using the same three evaluation metrics as mentioned above, namely joint trajectory tracking error, torque smoothness and mechanical energy consumption proxy. The results are presented in Table 4.

**Table 4:** Performance comparison of different temporal encoders.

Sequence encoder	Joint tracking error (°)	Torque smoothness (RMS)	Mechanical energy consumption proxy
BiLSTM-based encoder	2.63	0.66	11.17
Transformer-based encoder	2.79	0.74	11.58

As Table 4 shows, the BiLSTM encoder outperforms the Transformer encoder in all three evaluation metrics. Specifically, the joint trajectory tracking error of the BiLSTM model is 2.63°, lower than 2.79° of the Transformer model. In terms of torque smoothness, the RMS of BiLSTM is 0.66 while that of Transformer is 0.74, indicating that the former achieves better continuity and stability in control output. For the mechanical energy consumption proxy, BiLSTM achieves 11.17, also lower than 11.58 of Transformer. Overall, BiLSTM exhibits superior comprehensive performance in control accuracy, control smoothness, and energy efficiency. This result demonstrates that BiLSTM is more suitable for modeling the dynamic variations of neuromuscular signals in the task scenario of this study. The main reason is that the time-frequency feature sequences derived from preprocessed neuromuscular signals present strong local continuity and phase-transition characteristics, with stable evolutionary relationships between adjacent time windows. By leveraging the bidirectional recurrent structure, BiLSTM can more sufficiently extract the correlation information between consecutive time steps, thereby reconstructing motion variation trends and continuous motion intentions more accurately. In contrast, although Transformer has strong capability in modeling global sequence relationships, its advantage in global attention has not been translated into better closed-loop control performance under the current data scale and sequence length. Instead, it performs slightly worse than BiLSTM in terms of control smoothness and energy consumption metrics.

Furthermore, from the perspectives of training stability and control coupling, BiLSTM has a moderate parameter size, which enables it to cooperate more effectively with the subsequent DRL control strategy in the present task. Although the Transformer encoder can model temporal dependencies over a wider range, the exoskeleton control task driven by neuromuscular signals places more emphasis on motion trend variations, gait phase transitions, and short-term muscle activation patterns within local time windows. Therefore, the bidirectional local temporal modeling emphasized by BiLSTM is more consistent with the requirements of such tasks. Based on the comprehensive experimental results, it can be concluded that in the “signal–intention–control” framework constructed in this study, BiLSTM, as the motion intention modeling module, can provide more stable and control-valuable temporal representations.

## 6 Conclusion

In this study, an overall research flow based on open datasets and simulation platform is constructed, from neuromuscular signal processing, motion intention modeling to DRL control strategy design, forming a relatively complete “signal-intention-control” integrated framework. The study shows that reasonable intention modeling and reinforcement learning design can significantly improve the control accuracy and quality of exoskeleton, and maintain good robustness and generalization ability under the conditions of disturbance and task change. It provides a reproducible technical path and reference for the subsequent intelligent rehabilitation and algorithm design of exoskeleton. Although this study has achieved certain progress in the control of neuromuscular signal-driven exoskeletons, it still has limitations that require further improvement and expansion in subsequent work. The control algorithm is mainly based on a single Soft Actor-Critic framework. Although it performs well in continuous control tasks, there are still some shortcomings in training cost, sample efficiency and sensitivity to reward function design. At present, strategy training relies on large-scale interactive samples, which is time-consuming to adjust parameters and sensitive to reward weight setting and normalization. In the follow-up work, methods such as model reinforcement learning, off-line reinforcement learning or hierarchical reinforcement learning can be considered to improve sample efficiency and training stability, and at the same time reduce the dependence on artificial reward design. Generally speaking, this study provides a reproducible and extensible basic framework for DRL exoskeleton control driven by neuromuscular signals. The follow-up research will continue to expand in the directions of data diversity, algorithm efficiency, multimodal fusion and real system verification to further promote the development of intelligent exoskeleton control technology to a higher intelligent, adaptive, safe and reliable direction.

## Data Availability

The original contributions presented in the study are included in the article/supplementary material, further inquiries can be directed to the corresponding author.
